# Continuity of care and its associations with self-reported health, clinical characteristics and follow-up services after percutaneous coronary intervention

**DOI:** 10.1186/s12913-020-4908-1

**Published:** 2020-01-31

**Authors:** Irene Valaker, Bengt Fridlund, Tore Wentzel-Larsen, Jan Erik Nordrehaug, Svein Rotevatn, Maj-Britt Råholm, Tone M. Norekvål

**Affiliations:** 1 Faculty of Health and Social Sciences, Western Norway University of Applied Sciences, Svanehaugvegen 1, 6812 Førde, Norway; 2 Department of Heart Disease, Haukeland University Hospital, Box 1400, 5021 Bergen, Norway; 3 Centre for Interprofessional Collaboration within Emergency care (CICE), Linnaeus University, 351 95 Växjö, Sweden; 4 Centre for Clinical Research, Haukeland University Hospital, Haukelandsveien 28, 5009 Bergen, Norway; 5 Centre for Child and Adolescent Mental Health, Eastern and Southern Norway, Gullhaugveien 1-3, 0484 Oslo, Norway; 6 Norwegian Centre for Violence and Traumatic Stress Studies, Gullhaugveien 1, 0484 Oslo, Norway; 7 Department of Clinical Science, Faculty of Medicine, University of Bergen, P.O box 7804, 5020 Bergen, Norway; 8 Department of Cardiology, Stavanger University Hospital, Gerd-Ragna Bloch Thorsens gate 8, 4011 Stavanger, Norway; 9 Norwegian Registry for Invasive Cardiology, 5021 Bergen, Norway

**Keywords:** Percutaneous coronary intervention, Continuity of care, Self-reported health status

## Abstract

**Aims:**

Complexity of care in patients with coronary artery disease is increasing, due to ageing, improved treatment, and more specialised care. Patients receive care from various healthcare providers in many settings. Still, few studies have evaluated continuity of care across primary and secondary care levels for patients after percutaneous coronary intervention (PCI). This study aimed to determine multifaceted aspects of continuity of care and associations with socio-demographic characteristics, self-reported health, clinical characteristics and follow-up services for patients after PCI.

**Methods:**

This multi-centre prospective cohort study collected data at baseline and two-month follow-up from medical records, national registries and patient self-reports. Univariable and hierarchical regressions were performed using the Heart Continuity of Care Questionnaire total score as the dependent variable.

**Results:**

In total, 1695 patients were included at baseline, and 1318 (78%) completed the two-month follow-up. Patients stated not being adequately informed about *lifestyle* changes, medication and follow-up care. Those experiencing poorer health status after PCI scored significantly worse on continuity of care. Patients with ST-segment elevation myocardial infarction scored significantly better on informational and management continuity than those with other cardiac diagnoses. The regression analyses showed significantly better continuity (*P* ≤ 0.034) in patients who were male, received written information from hospital, were transferred to another hospital before discharge, received follow-up from their general practitioner or had sufficient consultation time after discharge from hospital.

**Conclusion:**

Risk factors for sub-optimal continuity were identified. These factors are important to patients, healthcare providers and policy makers. Action should be taken to educate patients, reconcile discharge plans and organise post-discharge services. Designing pathways with an interdisciplinary approach and shared responsibility between healthcare settings is recommended.

## Background

Modern cardiology has seen significant advancement in percutaneous coronary intervention (PCI) techniques and technology [1]. This ultimately means that more people survive, and patients have shorter hospital stays and return sooner to the community. In patients after PCI secondary prevention strategies such as risk factor management, lifestyle changes and pharmacological optimization are highly recommended [2]. As a result, hospital discharge is a critical moment for therapeutic recommendation and planning for secondary prevention and follow-up visits [1–3]. An extensive amount of information must be shared between healthcare settings which is a great challenge when taking care of the patients after PCI. This information includes medical history, diagnostics, laboratory, medication reconciliation and risk stratification [4]. Despite this fact, few studies have evaluated continuity of care across primary and secondary care levels for patients after PCI [5] .

Continuity of care has been garnering more attention in recent years [6] especially after Haggerty et. al’s [7] synthesis to develop a common understanding of the concept. The framework classifies continuity according to three domains: *informational* – the use of information on past events and personal circumstances to make current care appropriate for each individual, *relational* – an ongoing therapeutic relationship between a patient and one or more providers, and *management* – a consistent and coherent approach to the management of a health condition that is responsive to the patient’s changing needs [7]. In a systematic review, instruments measuring continuity of care were identified and the Heart Continuity of Care Questionnaire (HCCQ) was recommended for cardiac patients [8].

The association between continuity of care and patient-reported outcomes has been studied, but very few studies have scrutinised continuity of care from a multiple component perspective [6, 9, 10]. Cross-sectional studies analysing perceptions of the three domains of continuity between primary and secondary care found that healthcare area, age, educational level and comorbidity were related to overall perceptions of continuity of care [9, 10]. Some studies suggested differences related to age and educational level – the elderly population was more likely to perceive better continuity of care, whilst higher education was significantly associated with worse ratings [6, 10]. Additionally, there is some evidence that continuity of care is more important for patients with complex needs, and that patients with poor self-rated health are more critical to the care they receive [6, 11]. However, the influence of socio-demographic level, health status or gender is inconclusive in the different domains of continuity of care, and the significance of continuity of care attributed by specific patient groups varies [10].

General practitioners (GP) are the main coordinators of patients’ care in the community and assist patients through their transition from hospital to home [12]. Repeated contact with a single healthcare provider is linked with stronger relationships, better information transfer and more consistent management [6, 12]. Unfortunately, factors influencing continuity of care are not extensively studied for patients after PCI [3, 5, 13]. The aim of this study was therefore to determine multifaceted aspects of continuity of care and their associations with socio-demographic characteristics, self-reported health, clinical characteristics and follow-up services for patients after PCI.

## Methods

### Design and study population

The study, which is part of the prospective multicentre register-based CONCARD^PCI^ study [14], included patients from three centres from June 2017 throughout December 2018. Inclusion criteria were patients undergoing PCI, ≥18 years, and living at home at the time of inclusion. Exclusion criteria were not speaking Norwegian or unable to fill out the questionnaires due to reduced capacity, institutionalised patients and patients with an expected lifetime of less than 1 year. Additionally, patients undergoing PCI without stent implantation or undergoing PCI related to transcatheter aortic valve implantation or MitraClip were excluded, as were readmitted patients previously included in CONCARD^PCI^.

### Measurement

#### Socio-demographic and clinical characteristics

Socio-demographic characteristics included age, gender, cohabitation status, work status, educational level, duration of hospital stay, CR participation (planned, ongoing or completed) and follow-up with the GP. Disease-related outcomes included cardiac diagnosis, complications at hospital, clinical pathway (acute, sub-acute and planned), prior PCI, prior cardiac surgery, NYHA classification and comorbidity.

#### The heart continuity of care questionnaire (HCCQ)

The HCCQ is a 33-item self-report instrument used to assess three domains of perceived continuity, including informational (17 items), relational (10 items) and management (6 items) subscales, corresponding to the continuity of care model of Haggerty et al. [7]. From the patient perspective, the instrument covers major topics in cardiac care: heart condition explained, communication among healthcare providers, preparation for discharge, post-hospital care, post-hospital review of treatment, consistent information, information on medication, and knowledge on physical and dietary needs. Items were rated on a five-point.

Likert scale from 1 (strongly disagree) to 5 (strongly agree), with an additional category for “not applicable”. The half rule was used for missing data, i.e. using the mean of the answered items in the subscale, if at least half of that subscale was answered [15]. The HCCQ is a comprehensive, valid and reliable instrument for patients with congestive heart failure, atrial fibrillation and acute coronary syndrome [5, 13]. Recent psychometric testing showed that the instrument was satisfactory in the Norwegian context for patients after PCI [16].

#### The quality of life questionnaire abbreviated WHOQOL-BREF

WHOQOL-BREF includes a global measure of overall quality of life (QOL) and is applied in this study as the question “How would you rate your QOL?” WHO defines QOL as *“individuals’ perception of their position in life in the context of the culture and value systems in which they live, and in relation to their goals, expectations, standards and concerns”.* The item was rated on a five-point Likert scale from 1 (very poor) to 5 (very good). The instrument has acceptable psychometric properties in the Norwegian population [17, 18].

#### RAND 12-item short form health survey (RAND-12)

The 12-item generic self-report instrument was developed to reproduce the physical and mental component summary scores of the RAND-36 [19]. The RAND-12 has three to five response levels, with higher scores reflecting better self-reported health. Summary scores are standardised to a mean of 50 and a standard deviation of 10. The RAND-12 is a valid and reliable instrument when used in the Norwegian population [19, 20].

#### The myocardial infarction dimensional assessment scale (MIDAS)

The 35 items in MIDAS measure seven areas of health status and daily life change for patients with myocardial infarction. The self-report instrument covers seven topic areas: physical activity (12 items), insecurity (9 items), emotional reaction (4 items), dependency (3 items), concerns over medication (2 items) and side effects (2 items). Items were rated on a five-point Likert scale from 1 (never) to 5 (always). Each subscale is transformed from 0 to 100, with higher scores indicating a poorer health status. MIDAS appears to be a valid and reliable instrument showing trustworthy Cronbach’s alpha values (0.74–0.95) [21], and there is ongoing validation work in the Norwegian context to be published elsewhere.

### Data collection

All patients undergoing PCI at three large centres in Norway were prospectively screened for eligibility and included in the cohort study. Screening was performed in the hospital setting by the site coordinator and trained CONCARD^PCI^ study nurses. Daily admission records and operating programmes were reviewed to identify potentially eligible patients. Data on the included patients were collected from the patients’ paper and pencil self-report and from the Norwegian Registry for Invasive Cardiology (NORIC). Baseline self-reports were obtained after PCI, but before discharge from hospital. The self-administered instruments were then distributed by postal mail at the two-month follow-up. This time interval was chosen to ensure time for follow-up care so that the patients could provide an adequate evaluation of early post-discharge continuity of care. Two patient representatives with a history of coronary artery disease (CAD), and who were trained to be patient representatives both in healthcare and research settings, provided input to CONCARD^PCI^ [14].

### Data analysis

A descriptive analysis was conducted of the patients’ experiences of continuity of care, socio-demographic characteristics, self-reported health, clinical characteristics and follow-up services for patients after PCI. Item means, standard deviation and missing rates were calculated for the HCCQ. For comparisons between groups by *socio*-*demographic* and clinical characteristics, unpaired t-test and ANOVA were used for continuous variables and a chi-squared test for discrete variables. A paired t-test for RAND-12 scores and an exact marginal homogeneity test for WHOQOL-BREF were used to analyse the difference between scores at baseline and two-month follow-up. A post-hoc test was conducted using Tukey. Pearson correlations were used for continuous variables, while Spearman correlations were used for ordinal variables as appropriate. A strong correlation was operationally defined as *r* > 0.70, moderate to substantial as 0.30–0.70 and weak as < 0.30, in absolute value [22]. Hierarchical linear regression analysis was performed to determine the relationship between continuity of care as the dependent variable and individual factors, health-related factors and healthservice factors. A multivariate Wald test was used for multi-part categorical explanatory variables for calculating the overall *P*-value. Multiple imputation, with 200 imputed data sets, was used to estimate the regression models [23]. The variance inflation factor (*VIF*) was used to assess multicollinearity between predictors in complete case analyses, with VIF greater than 10 regarded as an indication of substantial multicollinearity. Based on VIF, the variable “sufficient time in consultations with GP”, showed substantial multicollinearity and the three first categories were merged in the regression analysis, resulting in VIF ≤ 6.15 in all regression analyses. To assess the goodness of fit R-squared (R^2^) were calculated. Statistical software SPSS (IBM Corp. Released 2016. IBM SPSS Statistics for Windows, Version 24.0. Armonk, NY: IBM Corp.) was used for most analyses. For the hierarchical regression analyses, R (The R Foundation for Statistical Computing, Vienna Austria) was used, with VIF calculations using the function ols_vif_tol in the R package R package olsrr, and multiple imputation using the R package mice, with the mice function D1 used for Wald tests.

## Results

### Socio-demographic and clinical characteristics

**Fig. 1:**
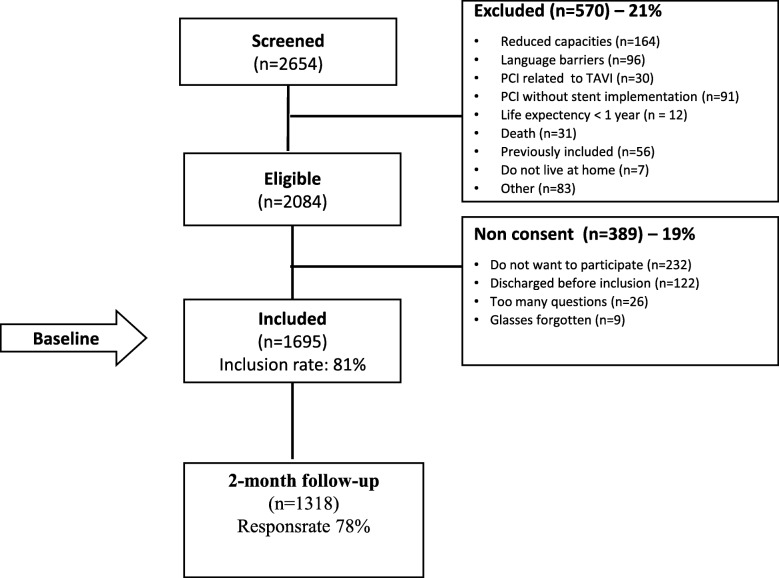
Flowchart

In total, 1695 patients were included at baseline and of those, 1318 (78%) completed the two-month follow-up. In Fig. 1, a flowchart presents the overall number of patients. Socio-demographic, clinical characteristics and patient-reported variables of patients after PCI are presented in Table 1. More than three quarters of patients were men and the mean age was 66 years. About one-fifth was diagnosed with ST-segment elevation myocardial infarction (STEMI) and more than three-quarters of the patients were discharged directly to their homes.

**Table 1:** Socio-demographic, clinical characteristics and patient-reported variables of patients after percutaneous coronary intervention^a^ (*n* = 1695)

Study population	Baseline n (%) or Mean (SD)	Two-month follow-up n (%) or Mean (SD)
Gender (male)	1313 (77.5)	1025 (77.8)
Age in years (mean, SD)	65.8 (10.9)	66.5 (10.5)
Cohabiting	1119 (77.9)	960 (80.3)
Education level		
Primary school	330 (22.1)	270 (21.8)
Trade school	536 (35.9)	445 (35.9)
High school	151 (10.1)	121 (9.8)
College/university, less than 4 years	259 (17.3)	212 (17.1)
College/university	218 (14.6)	191 (15.4)
Employed		
Full-time work	489 (32.3)	389 (31.0)
Retired	759 (50.1)	663 (52.8)
Disability pension	123 (8.1)	90 (7.2)
Other (e.g. part-time work, sick leave, seeking employment)	144 (9.5)	113 (9.0)
Coronary heart disease		
STEMI	336 (19.8)	255 (19.3)
NSTEMI	518 (30.6)	402 (30.5)
UAP	266 (15.7)	208 (15.8)
Stable coronary diseases	473 (27.9)	381 (28.9)
Other	102 (6.0)	72 (5.5)
Clinical pathway		
Acute	380 (22.4)	292 (22.2)
Sub-acute	768 (45.3)	594 (45.1)
Planned	547 (32.3)	432 (32.8)
Complication at hospital	32 (2.2)	23 (2.0)
Previous myocardial infarction	346 (20.5)	268 (20.4)
Previous PCI	426 (25.1)	324 (24.6)
Previous coronary bypass surgery	180 (10.6)	152 (11.5)
Previous stroke	72 (4.3)	51 (3.9)
Diabetes	314 (18.7)	221 (16.9)
Hypertension	911 (54.5)	708 (54.4)
Peripheral artery disease	129 (7.8)	96 (7.5)
NYHA classification		
I	161 (33.0)	131 (33.3)
II	265 (54.3)	216 (55.0)
III – IV	62 (12.7)	46 (11.7)
Current smokers	372 (23.8)	248 (20.5)
Duration of hospital stay		
1 day		248 (19.5)
2 days		209 (16.4)
3 days		219 (17.2)
4 days		230 (18.0)
More than 4 days		369 (28.9)
Transferred		
Discharged to home		881 (69.0)
Transferred to another hospital		347 (27.2)
Other		49 (3.8)
CR participation (planned, ongoing or completed)		518 (41.7)
Not offered CR as reason for not participating		387 (49.4)
First post-discharge meeting with GP		
Before 4 weeks		770 (61.0)
Within 4–8 weeks		332 (26.3)
Not visited the GP		160 (12.7)
Consultation with the regular GP at first appointment		
Yes		926 (84.0)
No, I was visiting a locum tenens physician/junior doctor		177 (16.0)
Sufficient time in consultations with GP		
Not at all		26 (2.0)
To a small extent		59 (4.6)
To some degree		283 (22.0)
To a large degree		648 (50.5)
To a very large degree		268 (20.9)

^a^Total counts (n) for a given variable may not necessarily sum to 1695 at baseline and 1317 at 2-month follow-up, because some patients failed to answer some items. *Abbreviations*: *PCI* percutaneous coronary intervention, *STEMI* ST-segment elevation myocardial infarction, *NSTEMI* Non–ST-segment elevation myocardial infarction, *UAP* unstable angina, *GP* general practitioner, *CR* cardiac rehabilitation, *NYHA* New York Heart Association

### Self-reported health and quality of life

A paired sample t-test showed that patients rated their QOL (measured with WHOQOL-BREF) worse after the two-month follow-up (mean difference = 0.19, *P* <  0.001). However, patients rated their general self-reported health (measured with RAND-12) better after the two-month follow-up in terms of both the mental component (mean difference = 1.56, *P* <  0.001) and physical component (mean difference = 2.15, *P* <  0.001). The disease-specific instrument (MIDAS) that measures health status and daily life changes showed a total score with mean 25.42 (SD = 15.78) at two-month follow-up. Patients scored less favourable on concerns of side effects and medication, physical activity, emotional reaction (Table 2).

**Table 2:** Self-reported health and quality of life of patients after percutaneous coronary intervention

Instrument	Baseline	Two-month follow-up	*P*-value
WHOQOL-BREF	*n* = 1498 Count (%)	*n* = 1290 Count (%)	<.001
Very poor	14 (0.9)	20 (1.6)	
Poor	66 (4.4)	78 (6.0)	
Neither good nor poor	271 (18.1)	296 (22.9)	
Good	858 (57.3)	716 (55.5)	
Very good	289 (19.3)	180 (14.0)	
RAND-12	*n* = 1289 Mean (SD)	*n* = 1159 Mean (SD)	<.001
Physical component, Mean (SD)	43.9 (10.8)	46.6 (10.7)	
Mental component, Mean (SD)	46.4 (11.1)	48.7 (10.9)	
MIDAS		*n* = 1253 Mean (SD)	
Total Mean (SD)		25.4 (15.8)	
Physical activity, Mean (SD)		27.8 (18.7)	
Insecurity Mean, Mean (SD)		19.4 (18.9)	
Emotional reaction, Mean (SD)		25.8 (20.2)	
Dependency, Mean (SD)		19.2 (18.7)	
Diet, Mean (SD)		24.2 (20.1)	
Concerns of medication, Mean (SD)		36.9 (26.6)	
Concerns of side effects, Mean (SD)		37.4 (27.0)	

*Abbreviations WHOQOL-BREF* World Health Organization Quality of Life, *RAND-12* Health Status Inventory; physical and mental component, *MIDAS* Myocardial Infarction Dimensional Assessment Scale. MIDAS has a range from 0 (best possible health as measured by the scale) through to 100 (worst health as measured by the scale). A paired t-test for RAND-12 scores and an exact marginal homogeneity test for WHOQOL-BREF were used to analyse the difference between scores at baseline and two-month follow-up

### Continuity of care

**Fig. 2:**
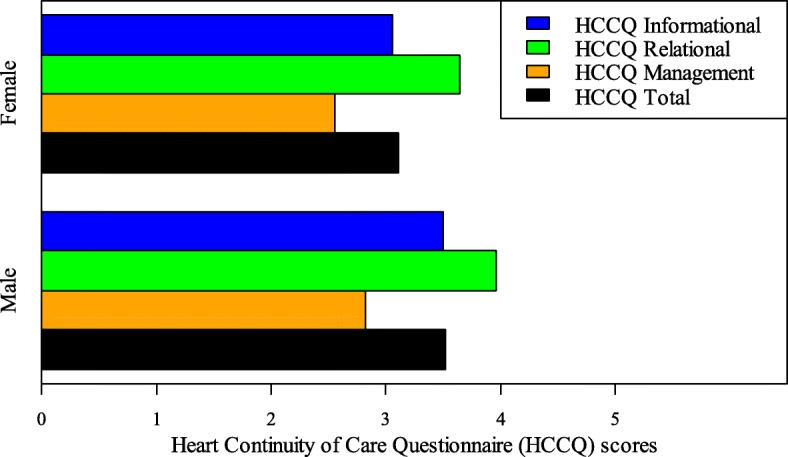
Total mean of the Heart Continuity of Care Questionnaire (HCCQ) and gender differences

Descriptive statistics of the 33 items of the HCCQ are presented in Table 3. Several items represent an area of concern, with a mean below 3.75 or a substantial proportion of patients rated 1 or 2, indicating negative care experiences [13]. For instance, 61% of the patients stated that they were not adequately informed about what to do if they experienced side effects and about 37% were not adequately informed about who to contact in the event of problems after discharge. Similarly, about 54% of patients reported that their physician had not adequately reviewed their treatment plan following discharge. The total mean of the HCCQ and gender differences are shown in Fig. 2. The red striped line shows the cut-off value, with scores below 3.75 indicating negative care experiences [13]. The total mean score for information continuity was 3.33 (SD = 0.91), for relational continuity 3.72 (SD = 0.87), and for management continuity 2.57 (SD = 1.28).

**Table 3:** Item analysis of the 33 items in the Heart Continuity of Care Questionnaire (HCCQ)

HCCQ item number and descriptions	n	Mean	SD	Strongly or somewhat disagree (%)	Not applicable
Informational continuity					
Provided with information	1291	4.06	1.10	11.7	5
Condition clearly explained	1282	4.24	1.06	9.6	7
Told what symptoms to expect	1239	**3.26**	1.33	30.3	34
Given opportunity to ask questions	1253	4.17	1.08	10.1	31
Medication explained.	1255	4.08	1.23	13.1	30
Told when and how to take medication	1251	4.49	0.99	6.8	29
Told about potential side effects	1255	**2.70**	1.37	48.8	28
Told what to do if side effects occurred	1256	**2.36**	1.31	61.2	31
Given same information about medications	1227	**3.47**	1.32	21.7	48
Told what changes to make to diet	1207	**2.56**	1.39	50.9	67
Instruction to plan own daily meals	1205	**2.52**	1.39	53.4	64
Explained influence on lifestyle	1220	**2.52**	1.34	52.9	57
Explained physical activity	1229	**2.62**	1.42	49.9	44
Providers communicated well in hospital	1233	4.11	1.03	5.8	45
Well prepared for discharge	1265	**3.44**	1.31	25.4	16
Told what symptoms to call doctor about	1252	**2.91**	1.44	41.9	25
Consistent information about symptoms to seek help for	1186	**2.97**	1.38	35.7	68
Relational continuity					
Providers communicated well in planning move	1232	3.97	1.15	10.1	47
Providers communicated well after discharge	1111	**3.46**	1.19	15.7	149
Providers obtained needed information from other providers	1144	3.88	1.06	6.6	105
Family physician involved in care	1203	**3.45**	1.41	24.9	69
Knew who to contact about problems after discharge	1226	**3.19**	1.58	36.6	41
Satisfied with care after discharge	1170	3.95	1.22	12.5	101
After discharge, could access services	1078	**3.59**	1.35	19.3	185
Doctor is aware of blood test results	1248	4.29	1.08	6.4	37
Consistent information from doctors	1142	**3.67**	1.30	16.0	112
Consistent information from doctors and other providers	1113	**3.59**	1.29	16.8	127
Management continuity					
Reviewed treatment plan	1148	**2.61**	1.63	53.5	107
Regularly scheduled appointments	1171	**3.13**	1.71	40.1	97
Reviewed heart medication	1222	**3.02**	1.75	44.2	56
Explained again how medication should be taken	1197	**2.72**	1.70	50.9	72
Explained again potential side effects	1193	**2.04**	1.40	69.9	72
Explained again what to do about side effects	1193	**1.96**	1.36	71.8	74

Scores range from 1 to 5 with higher scores denoting more positive continuity experiences. Items in bold represents an area of concern (mean less than 3.75). Patients had the option to choose “not applicable”, for example a patient who did not receive services following discharge would choose this category. This category is not included in the denominator in the computation of percentages Strongly or somewhat disagree

### Factors associated with perceived continuity of care

Table 4 presents group statistics and correlations between HCCQ domains and individual factors, health-related factors and healthservice factors. As shown, females were more likely to report appreciably worse on continuity of care in all continuity domains. Those cohabiting scored substantially better on information and relational continuity. Moreover, patients who received written patient information from hospital and who participated in CR scored significantly better in all continuity domains. Patients with an acute clinical pathway scored significantly better on the three continuity of care domains than planned pathways. STEMI patients scored significantly better on informational and management continuity of care than Non-ST-segment elevation myocardial infarction (NSTEMI), stabile coronary syndrome and unstable angina patients (*P* ≤ 0.011). Moreover, STEMI patients scored better on relational continuity than stable coronary diseases (*P* = 0.006). Table 4 shows a weak negative correlation between informational and management continuity and age (*r* = 0.063, *r* = 0.090). There were also weak to moderate positive correlations between continuity of care and duration of hospital stay (*r* = 0.061–0.166) and sufficient time in consultation with GP (*r* = 0.191–0.364). Moreover, a weak positive correlation existed between continuity of care and a global measure of overall QOL (*r* = 0.114–0.234), generic self-report health (*r* = 0.065–0.211) and disease-specific health status (*r* = 0.073–0.255).

**Table 4:** Group statistics and correlations between Heart Continuity of Care Questionnaire (HCCQ) domains and individual factors, health-related factors and health service factors

Variable	Informational continuity Mean difference (95% CI) *P*-value	Relational continuity Mean difference 95% CI *P*-value	Management continuity Mean difference 95% CI *P*-value
Gender (male)	0.45 (0.32, 0.57) *P* < 0.001	0.30 (0.17,0.43) *P* < 0.001	0.28 (0.11,0.45) *P* = 0.001
Cohabiting	0.24 (0.10, 0.38) *P* = 0.001	0.20 (0.06, 0.33) *P* = 0.005	0.11 (−0.08, 0.30) *P* = 0.250
Written patient information from hospital	0.58 (0.44, 0.72) *P* < 0.001	0.57 (0.44, 0.71) *P* < 0.001	0.71 (0.53, 0.89) *P* < 0.001
Transferred to another hospital after PCI	0.12 (0.01, 0.24) *P* = 0.033	0.16 (0.05, 0.27) *P* = 0.004	0.35 (0.19, 0.52) *P* < 0.001
Consultation with the regular GP at first appointment	0.26 (0.11, 0.42) *P* = 0.001	0.42 (0.27, 0.58) *P* < 0.001	0.36 (0.16, 0.56) *P* = 0.001
CR participation (planned, ongoing or completed)	0.15 (0.05, 0.25) *P* = 0.004	0.24 (0.14, 0.34) *P* < 0.001	0.42 (0.28, 0.57) *P* < 0.001
Comorbidity	0.16 (0.05,0.26) *P* = 0.002	0.06 (−0.04,0.16) *P* = 0.279	0.13 (− 0.02,0.28) *P* = 0.091
Complication at hospital	−0.06 (− 0.45, 0.32) *P* = 0.738	0.06 (− 0.32, 0.44) *P* = 0.740	0.27 (− 0.28, 0.82) *P* = 0.326
Variable	Informational continuity *P*-value between groups Mean (SD)	Relational continuity *P*-value between groups Mean (SD)	Management continuity *P*-value between groups Mean (SD)
Coronary heart disease ^a^	*P* < 0.001	*P* = 0.008	*P* < 0.001
STEMI	3.57 (0.84)	3.88 (0.80)	3.02 (1.23)
NSTEMI	3.32 (0.94)	3.72 (0.88)	2.59 (1.26)
UAP	3.24 (0.92)	3.66 (0.90)	2.52 (1.30)
Stable coronary diseases	3.24 (0,90)	3.62 (0.88)	2.26 (1.24)
Other	3.37 (0.85)	3.79 (0.81)	2.67 (1.19)
Clinical pathway ^b^	*P* < 0.001	*P* < 0.008	*P* < 0.001
Acute	3.56 (0.82)	3.85 (0.80)	3.00 (1.23)
Sub-acute	3.29 (0.94)	3.71 (0.88)	2.57 (1.26)
Planned	3.24 (0.89)	3.64 (0.88)	2.29 (1.25)
First post-discharge meeting with GP ^c^	*P* = 0.055	*P* < 0.001	*P* < 0.001
Before 4 weeks	3.35 (0.93)	3.79 (0.84)	2.76 (1.25)
Within 4–8 weeks	3.40 (0.86)	3.78 (0.83)	2.63 (1.24)
Not visited the GP	3.19 (0.91)	3.28 (0.89)	1.74 (1.10)
Variable	Informational continuity Correlation (*P*-value)	Relational continuity Correlation (*P*-value)	Management continuity Correlation (*P*-value)
Age	−0.063 (0.025)	−0.038 (0.184)	−0.090 (0.002)
Education level	0.022 (0.452)	0.023 (0.425)	0.006 (0.836)
Duration of hospital stay	0.065 (0.023)	0.061 (0.035)	0.166 (< 0.001)
Sufficient time in consultations with GP	0.191 (< 0.001)	0.364 (< 0.001)	0.273 (< 0.001)
MIDAS (two-month follow-up)	−0.255 (< 0.001)	− 0.217 (< 0.001)	− 0.073 (0.012)
WHOQL-BREF (two-month follow-up)	0.234 (< 0.001)	0.203 (< 0.001)	0.114 (< 0.001)
RAND-12 (two-month follow-up)			
Mental component	0.206 (< 0.001)	0.065 (0.031)	0.188 (< 0.001)
Physical component	0.211 (< 0.001)	0.191 (< 0.001)	0.095 (0.002)

*Abbreviations PCI* percutaneous coronary intervention, *GP* general practitioner, *CR* cardiac rehabilitation, *STEMI* ST-segment elevation myocardial infarction, *NSTEMI* Non–ST-segment elevation myocardial infarction, *UAP* unstable angina, *MIDAS* Myocardial Infarction Dimensional Assessment Scale, *WHOQL* World Health Organization Quality of Life, *RAND-12* Health Status Inventory; physical and mental component

Hypotheses about possible relationships between patient characteristics and domain scores on the HCCQ

^a^STEMI patients scored significantly better than NSTEMI, UAP and stabile coronary diseases on informational and management continuity of care (*P* = 0.008–0.001)

^b^Acute clinical pathway scored significantly better than planned on the three continuity of care domains (*P* < 0.005) and acute clinical pathway scored significantly better than acute and sub-acute on management continuity of care (*P* < 0.002)

^c^Patients who had not visited their GP scored significantly worse than those who saw their GP before 4 weeks or within 4–8 weeks (*P* < 0.001)

The hierarchical linear regression analysis for perceptions of total continuity of cardiac care at two-month follow-up is reported in Table 5. The analyses utilized all available information for the 1267 patients with complete scores on the total HCCQ score. There were some differences compared with complete case analyses, as expected the precision was better when multiply imputed data were used. Gender, written patient information, discharged to another hospital after PCI, follow-up with GPs after discharge and consultation time were significant predictors. Adjusted R squared for Block 1 = 0.039, Block 2 = 0.063 and Block 3 = 0.220.

**Table 5:** Hierarchical linear regression analysis with predictors associated with perceptions of continuity of care at the two-month follow-up (*n* = 1267)

Variables	Unadjusted regression			Block 1			Block 2			Block 3		
	coef.	CI (95%)	*p*	coef	CI (95%)	*p*	coef	CI (95%)	*p*	coef	CI (95%)	*p*
Block 1: Individual factors												
Male Gender	0.390	(0.279, 0.500)	< 0.001	0.364	(0.249, 0.479)	< 0.001	0.344	(0.226, 0.463)	< 0.001	0.318	(0.208, 0.428)	< 0.001
Age in years	− 0.006	(−0.011, − 0.002)	0.008	− 0.004	(− 0.008, 0.001)	0.094	− 0.003	(− 0.008, 0.002)	0.185	− 0.001	(− 0.005, 0.004)	0.779
Not living alone	0.201	(0.078, 0.324)	0.001	0.130	(0.005, 0.255)	0.041	0.116	(−0.008, 0.241)	0.068	0.087	(−0.029, 0.202)	0.142
Education level			0.845			0.686			0.409			0.585
Primary School (ref.)												
Trade school	0.037	(−0.096, 0.170)	0.590	−0.061	(− 0.194, 0.073)	0.375	− 0.072	(− 0.206, 0.062)	0.291	− 0.019	(− 0.143, 0.105)	0.760
High School	0.080	(−0.106, 0.266)	0.401	0.020	(−0.164, 0.204)	0.833	−0.001	(−0.185, 0.183)	0.992	0.054	(−0.117, 0.225)	0.533
College/University	0.045	(−0.089, 0.180)	0.507	−0.053	(− 0.187, 0.082)	0.445	−0.103	(− 0.241, 0.035)	0.143	− 0.052	(− 0.180, 0.075)	0.420
Block 2: Health-related factors												
RAND-12 Physical (baseline)	0.010	(0.005, 0.015)	< 0.001				−0.004	(− 0.013, 0.005)	0.405	− 0.006	(− 0.015, 0.002)	0.151
RAND-12 Mental (baseline)	0.010	(0.006, 0.015)	< 0.001				0.008	(−0.001, 0.016)	0.093	0.007	(−0.001, 0.015)	0.098
Coronary heart disease			< 0.001						< 0.001			0.034
Stabile coronary diseases (ref.)												
UAP	0.059	(−0.086, 0.203)	0.426				0.081	(−0.062, 0.224)	0.268	−0.033	(− 0.186, 0.120)	0.672
NSTEMI	0.142	(0.022, 0.262)	0.021				0.142	(0.021, 0.262)	0.021	−0.030	(−0.174, 0.114)	0.679
STEMI	0.390	(0.254, 0.525)	< 0.001				0.338	(0.195, 0.481)	< 0.001	0.143	(−0.029, 0.315)	0.103
Other	0.217	(0.005, 0.429)	0.044				0.210	(− 0.000, 0.419)	0.050	0.164	(−0.040, 0.367)	0.115
Comorbidity DHPS	−0.121	(− 0.217, − 0.025)	0.014				−0.046	(− 0.148, 0.055)	0.372	−0.045	(− 0.138, 0.049)	0.349
Smoke			0.547						0.360			0.269
Never smoke (ref.)												
Smoked before >1mnd	−0.061	(−0.171, 0.049)	0.276				−0.058	(− 0.169, 0.053)	0.303	−0.084	(−0.186, 0.018)	0.106
Smoked	−0.043	(−0.178, 0.093)	0.535				−0.100	(−0.245, 0.044)	0.174	−0.049	(− 0.182, 0.083)	0.465
Previous PCI	−0.130	(− 0.238, − 0,022)	0.018				−0.075	(− 0.188, 0.037)	0.190	− 0.044	(−.0.149, 0.060)	0.405
Previous Coronary bypass surgery	−0.042	(− 0.188, 0.104)	0.573				0.036	(−0.116, 0.189)	0.638	0.048	(−0.092, 0.189)	0.500
Block 3: Healthservice factors												
Duration of hospital stay			< 0.001									0.369
1 day (ref.)												
2 days	0.209	(0.050, 0.368)	0.010							0.150	(0.001, 0.298)	0.049
3 days	0.277	(0.121, 0.433)	< 0.001							0.087	(−0.078, 0.252)	0.303
4 days	0.277	(0.124, 0.430)	< 0.001							0.055	(−0.118, 0.229)	0.531
More than 4 days	0.262	(0.124, 0.400)	< 0.001							0.058	(−0.104, 0.220)	0.482
Benefit from the written patient information from hospital			< 0.001									< 0.001
No (ref.)												
Yes	0.454	(0.312, 0.597)	< 0.001							0.344	(0.208, 0.481)	< 0.001
I don’t know	0.190	(0.024, 0.355)	0.024							0.100	(−0.057, 0.256)	0.211
Did not receive	− 0.164	(− 0.332, 0.003)	0.054							−0.121	(−0.278, 0.036)	0.131
Discharged to home	−0.185	(−0.291, − 0.080)	< 0.001							−0.116	(−0.224, − 0.009)	0.034
First post-discharge meeting with GP			< 0.001									0.068
Before 4 weeks (ref.)												
Within 4–8 weeks	−0.014	(−0.124, 0.096)	0.801							−0.001	(− 0.102, 0.100)	0.985
Not visited the GP	−0.446	(−0.591, − 0.301)	< 0.001							−0.185	(−0.347, − 0.023)	0.025
Consultation with the regular GP at first appointment	0.401	(0.281, 0.521)	< 0.001							0.191	(0.057, 0.326)	0.005
Enough time in consultations with GP			< 0.001									< 0.001
To some degree and less (ref.)												
To a large degree	0.476	(0.371, 0.581)	< 0.001							0.379	(0.276, 0.482)	< 0.001
To a very large degree	0.698	(0.569, 0.828)	< 0.001							0.558	(0.430, 0.686)	< 0.001
CR participation (planned, ongoing or completed)	0.224	(0.128, 0.320)	< 0.001							0.093	(−0.004, 0.190)	0.060
				Block 1 R^2^ = 0.043 Adjusted R Squared = 0.039			Block 2 R^2^ = 0.076 Adjusted R Squared = 0.063			Block 3 R^2^ = 0.239 Adjusted R Squared = 0.220		

Dependent Variable Heart Continuity of Care questionnaire (HCCQ), All analyses in the table are estimated using multiply imputed data for the 1267 observations with complete data for the dependent variable. *Abbreviations PCI* percutaneous coronary intervention, *STEMI* ST-segment elevation myocardial infarction, *NSTEMI* Non–ST-segment elevation myocardial infarction, *UAP* unstable angina, *GP* general practitioner, *CR* cardiac rehabilitation, *WHOQL* World Health Organization Quality of Life, *RAND-12* Health Status Inventory, physical and mental component, *Comorbidity DHPS* diabetes, hypertension, peripheral artery disease and previous stroke

## Discussion

This study shows that patients after PCI report challenges concerning seamless flow of information and effective communication between hospital and community settings. Moreover, socio-demographic and clinical characteristics, such as gender, cardiac diagnosis, follow-up with GP and CR, influenced certain domains of continuity.

### Patient perception of continuity of care

Acute hospitalisation for CAD represents a significant event in a patient’s life [24]. According to an item analysis of the HCCQ, patients were not adequately informed about what symptoms to expect and the influence on lifestyle. Nor were they adequately informed about potential medication side effects and what to do in the event of side effects. Patients also lacked sufficient information about physical activity and dietary advice.

The European Society of Cardiology guidelines recommend implementing strategies for prevention, including lifestyle changes, risk factor management and pharmacological optimisation before hospital discharge to lower the risk of mortality and morbidity [2]. Teaching is an essential component of information continuity and recommendations for improving teaching emphasise a patient-centred approach in which the content and method of teaching are individualised, rather than the more typical approach of distributing standardised information based on diagnosis [25]. In addition to medical treatment, patients need to know what is wrong or how to stay well, what is likely to happen and how the cardiac diseases will affect them, in a language they understand [26]. However, most patients do not receive treatment according to standard guidelines for secondary prevention [4, 27]. The short hospital stay that is common in modern cardiac care makes it difficult to conduct inpatient education and training [3]. In the current study, more than half of the patients stayed at hospital for 3 days or less. As a result, integration and designed pathways between acute care and follow-up in the community are essential to ensuring that care is connected and coherent [4, 7].

There were patients who felt that healthcare providers did not communicate well with each other when planning the hospital discharge. Creating explicit management plans to ensure consistency during treatment is a recurrent theme in management continuity and depends on the receipt of informative discharge summaries from medical specialists [7]. However, previous research indicates a need for more effective communication, collaboration and teamwork [4, 9, 28–30]. Instead, each discipline and type of organisation tends to defend its authority at the expense of the overall healthcare system – a problem known as sub-optimisation [26]. Suggestions to achieve better integration between healthcare settings include clarifying responsibility and improving the implementation of technology, such as computer links and e-mails [3, 31].

Relational continuity between patients and healthcare providers is highly valued in primary care [32]. The HCCQ does not measure the strength of interpersonal relationships with healthcare providers and focuses on contact with the GP. Nevertheless, team-based care delivery, such as assigning GPs and nurses as key persons, is suggested to improve integration and provide long-term follow-up [33]. Communication knowledge and skills make this possible, and a positive interaction enhances patients’ ability to cope with illness and adhere to recommended lifestyle changes [4]. Patients in this study reported that their GPs were not adequately involved in their care and not all patients knew which healthcare provider to contact if problems arose post-discharge. In this respect, it seems important to understand potential threats to patient–healthcare communication as system barriers to adequate healthcare.

### Individual factors associated with perceived continuity of care

With regard to individual factors, older patients reported worse on continuity of care.

Older patients tend to be more vulnerable in the context of acute care and need extra professional help to navigate in a complex healthcare system [5, 9]. The environment and the routines in the hospital might be overwhelming and the transition out of the hospital stressful [34]. Patients living alone scored worse on informational and relational continuity of care. One explanation for this is that family members and significant others may have an impact on patients’ experience by helping them to remember medical information and follow-up treatment regimens [5].

This study found that female patients scored significantly worse on continuity than their male counterparts in all domains. The evidence of the influence of gender is inconclusive and varies between countries and diagnoses [9]. However, female patients reported fewer positive experiences in hospital care, particularly with respect to communication about medicines and discharge information [35]. Females have been at a higher risk of adverse cardiac events after PCI, compared with males. In addition, women are less likely to be referred for revascularisation for CAD and receive fewer guideline-recommended therapies [36, 37]. On the basis of these findings, healthcare providers should pay more attention to female patients in clinical practice to ensure continuity of care.

### Health-related factors associated with perceived continuity of care

Patients rated their QOL worse 2 months after discharge, and there was a correlation between QOL and all continuity of care domains. A possible explanation is that the majority of patients after PCI feel that they are back to normal soon after the treatment, leading them to view their illness as an acute event cured by the treatment, rather than an acute marker for a long-term condition [38].

MIDAS encompasses health and lifestyle changes specifically relevant to patients with CAD. Patients reported physical complaints, as well as concerns over medication and side effects. Patients who experienced greater continuity of care felt healthier and had fewer symptoms.

This is plausible because patients with worse health status will likely interact more frequently with the healthcare system [5, 6, 12, 39]. This suggests that healthcare providers need to be more attuned to the patients’ perceptions of the consequences of their cardiac disease and their need for more intensive integration [5].

This study shows that patients with comorbidity scored worse on informational continuity of care than those with just one health condition. Patients with more complex cardiac diseases may interact more frequently with the healthcare system and are likely to be particularly vulnerable to breaks in continuity of care. This is typically when patients are being passed between healthcare providers who do not communicate with each other [6, 9, 29]. On the other hand, the study found no indications that patients with complications after PCI scored less on continuity of care. The use of stents and aggressive antiplatelet therapy have led to a decreasing risk of major acute complications of PCI [1].

### Health service factors associated with perceived continuity of care

Clinical pathways and urgency levels differ based on the different clinical manifestations of CAD, and on whether procedures are performed in either emergent, planned or rescue situations [40]. The current study shows that patients with STEMI scored significantly better on informational and management continuity than those with other cardiac diagnoses. One explanation is related to the speed of treatment delivery, and was confirmed by the fact that those experiencing acute clinical pathways score better on continuity of care. Primary PCI is the first-line treatment for patients with STEMI, and centres providing primary PCI services maintain an infrastructure that enables them to perform at high standards of safety and efficacy. In contrast, patients with non-STEMI or unstable angina who are clinically unstable have an angiography (followed by PCI if indicated) within 24 h of becoming clinically unstable. This means that patients must wait at their local hospital before being transported to the PCI centre. These patients therefore experience more complex clinical pathways and are discharged sooner from hospital as compared to STEMI patients [41]. This is also consistent with the finding that patients who stayed in hospital for a longer period or were transported to another hospital before discharge experienced greater continuity of care. This gives healthcare providers more time when organising patient care as compared to patients with other CAD diagnoses.

A previous study found that one of the most consistently associated organisational factors was the consistency of healthcare providers [9]. However, the current study shows that 13% had not visited their GP 2 months post-discharge and scored significantly worse in all continuity of care domains. Moreover, 16% of the patients had their first consultation post-discharge with a locum tenens physician/junior physician rather than their own GP. These patients also scored significantly worse in all domains of continuity of care. Consulting more than one GP can initiate disorganised treatment plans or mean that patients are given different recommendations to follow [42]. Patients living in rural areas have limited local access to healthcare systems in their community, and many Norwegian municipalities are small and lack sufficient resources and competence [43]. Another important aspect identified was that not having enough consultation time with the GP post-discharge showed a negative correlation with all continuity domains. In today’s healthcare system, consultations are often delayed or rushed [26]. However, with increased emphasis on value and efficiency in healthcare delivery, sufficient time for conversation between healthcare providers and patients is an increasingly valuable resource.

Patients after PCI are recommended to participate in CR to improve patient outcomes [1, 4]. The CR enrolment process is dependent on patients being informed about CR by a healthcare provider, and the referred patient must then attend an intake assessment and can ultimately participate in the programme. A recent Norwegian study reported a participation rate varying from 20 to 31% among four regional health authorities [44]. In this study, 42% responded positively to the question on CR (planned, ongoing or completed). Patients who engaged in CR had better scores in continuity of care. When patients were asked why they were not participating, 49% had not been offered CR. The reasons for poor referral and participation are complex and multifactorial, and certain groups such as the elderly and females are shown to be less likely to participate [45]. Moreover, research indicates regional differences in CR participation, which is due to both lower availability of CR and longer travelling distances to locations offering these programmes [4, 44]. However, automated referral systems and patient education given by GPs and other healthcare providers regarding the benefits of CR are the most effective strategies for improving participation rates [4]. The use of modern technologies also offers interesting prospects for CR delivery [31].

### Methodological issues

Bias originates in the design stage of the study, such as in sample selection, or in data collection or analysis. However, CONCARD^PCI^ [14] has prioritized good planning of the study protocol and adequate sample size to avoid random errors substantially influencing the results of the study. Data were collected at baseline and at two-month follow-up to determine the relationship between continuity of care and other variables of interest. Although the response rate at two-month follow-up was high (78%), non-responders might represent a limitation. This type of design is limited in its ability to draw valid conclusions on causality and runs the risk of recall bias. Patients are the only ones who are able to experience whether care is connected and coherent over time, but self-report is dependent on honesty and that socially desirable answers are not generated. The HCCQ has proven to be a good instrument for patients after PCI in a Norwegian context, although the psychometric properties need to be further evaluated [16]. Finally, this study had a number of strengths including the large sample size and low refusal rate at two-month follow-up.

## Conclusions and implications

As patients after PCI move between hospital and community, the potential for discontinuity arises, and the healthcare system needs to take more responsibility to educate and counsel patients, reconcile discharge plans and organise post-discharge services. Predictors of total continuity of care were gender, diagnosis, follow-up with GPs and sufficient consultation time. A greater focus on subgroups of patients at high risk of discontinuity and factors associated with good continuity of care are essential. Whether poor continuity leads to worse patient outcomes, including (avoidable) hospital readmissions and mortality is a path for future research. Changes are required in the structures and processes of healthcare delivery, such as implementing team structures in primary care, supportive information systems and interactive technologies.

## Data Availability

Data can not be made available due to patient confidentiality reasons. Analysis files (R scripts, SPSS syntaxes, other) can be made publicly available from the PI upon reasonable request.

## Abbreviations

CAD: Coronary artery disease
CR: Cardiac rehabilitation
GP: General practitioner
HCCQ: Heart Continuity of Care Questionnaire
MIDAS: The Myocardial Infarction Dimensional Assessment Scale
NORIC: Norwegian Registry for Invasive Cardiology
NSTEMI: Non-ST-segment elevation myocardial infarction
PCI: Percutaneous coronary intervention
QOL: Quality of life
R^2^: R-squared
RAND-12: RAND 12-Item Short Form Health Survey
STEMI: ST-segment elevation myocardial infarction
VIF: Variance inflation factor
WHOQL: World Health Organization Quality of Life
